# Sjögren’s Disease-Associated Parotid MALT Lymphoma: Diagnostic Pitfall of Ultrasound-Guided Core Needle Biopsy

**DOI:** 10.3390/diagnostics16183067

**Published:** 2026-09-21

**Authors:** Shiou-Yi Chen, Wei-Chen Hung, Ping-Chia Cheng, Wu-Chia Lo, Chih-Ming Chang, Ming-Hsun Wen, Andy Wei-Ge Chen, Li-Jen Liao

**Affiliations:** 1 Department of Otolaryngology Head and Neck Surgery, Far Eastern Memorial Hospital, New Taipei City 220216, Taiwan; 2 Head and Neck Cancer Surveillance & Research Group, Far Eastern Memorial Hospital, New Taipei City 220216, Taiwan; 3 Department of Electrical Engineering, Yuan Ze University, Taoyuan 320315, Taiwan

**Keywords:** Sjögren’s disease, MALT lymphoma, parotid gland, ultrasound, core needle biopsy, diagnostic pitfall

## Abstract

Sjögren’s disease (SD) is a chronic autoimmune disease associated with an increased risk of extranodal marginal zone lymphoma of mucosa-associated lymphoid tissue (MALT lymphoma), particularly in the parotid glands. However, distinguishing benign SD-associated sialadenitis from early lymphoma remains a diagnostic challenge, especially when biopsy samples are limited. We report an elderly woman presenting with progressive bilateral parotid swelling accompanied by xerostomia and xerophthalmia. Serological testing revealed positive anti-SSA and anti-SSB antibodies, hypergammaglobulinemia, and hypocomplementemia, suggesting Sjögren syndrome. Neck ultrasound demonstrated multiple hypoechoic nodules in both parotid glands. Ultrasound-guided core needle biopsy (US-CNB) revealed lymphoepithelial sialadenitis with mixed lymphocytic infiltration suggestive of SD. Subsequent computed tomography demonstrated necrotic lymphadenopathy in the right parapharyngeal space. Surgical excision of the right parotid tumor was therefore performed, and histopathology confirmed extranodal marginal zone B-cell lymphoma (MALT lymphoma). Staging PET-CT revealed multi-site involvement. Following chemotherapy, a complete metabolic response was achieved, and the patient remained in remission during 30 months of follow-up. Careful surveillance is essential in patients with SD due to the increased risk of lymphoma. Repeat biopsy or surgical excision should be considered when imaging and clinical findings are discordant with CNB results.

**Figure 1:**
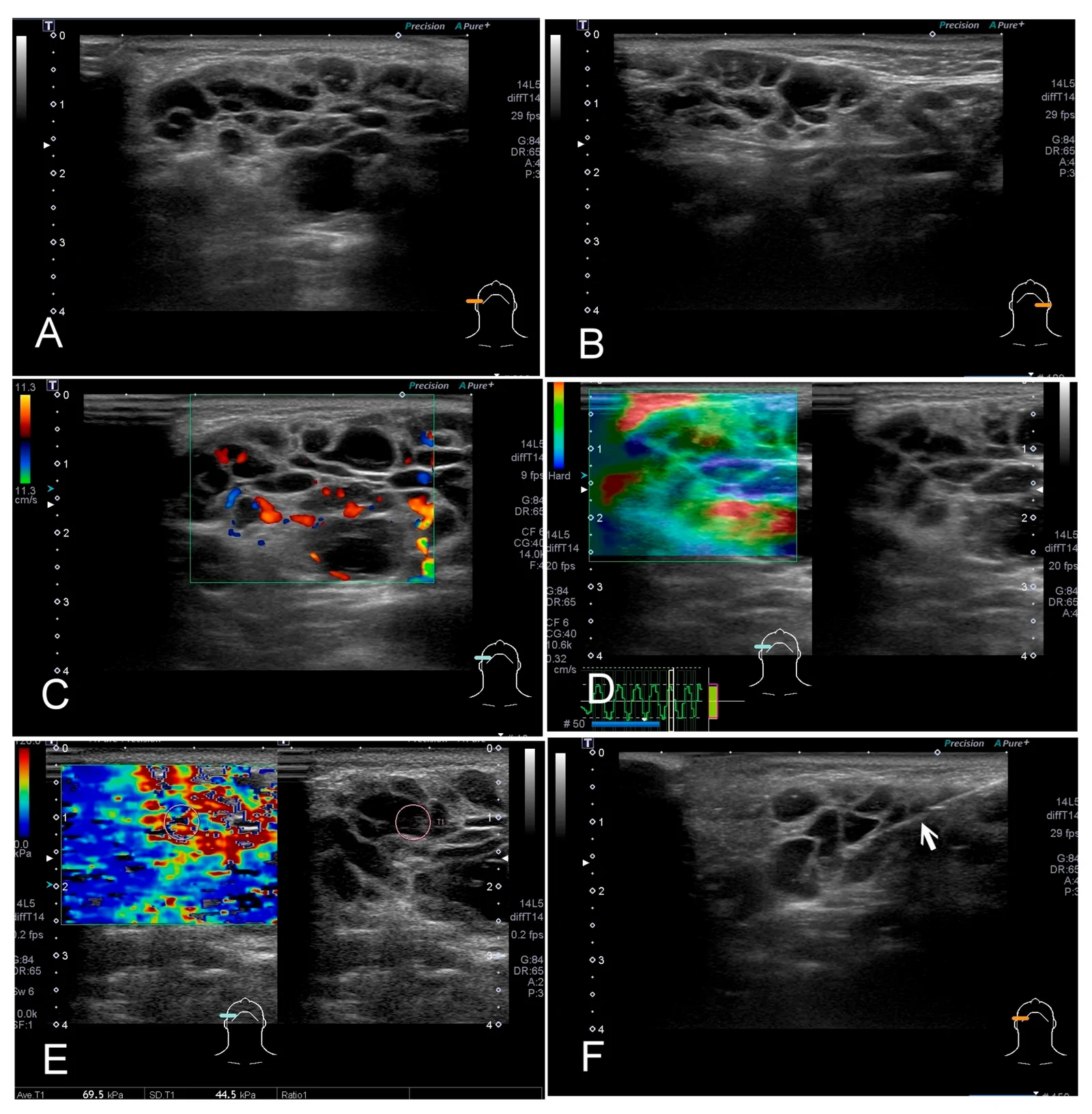
An elderly woman presented with a one-year history of progressive bilateral parotid gland swelling. The swelling was painless and more prominent on the right side. She also reported xerostomia and xerophthalmia, although the exact duration of these sicca symptoms was not clearly documented in the available medical records. She denied dysphagia, dyspnea, odynophagia, or voice changes. Her past medical history was unremarkable. She had previously undergone surgery for a benign liver cyst. Complete blood count revealed mild leukopenia (white blood cell count, 3.61 × 10^3^/μL) and mild anemia (hemoglobin, 10.6 g/dL; hematocrit, 34.1%), with a platelet count of 149 × 10^3^/μL. Biochemical testing showed hypokalemia (serum potassium, 3.0 mmol/L), while renal function, liver function, and lactate dehydrogenase levels were within the reference ranges. C-reactive protein was also within the institutional reference range. Autoimmune serological testing revealed positive anti-SSA (Ro) and anti-SSB (La) antibodies together with hypocomplementemia (C3, 75.3 mg/dL; C4, <8.0 mg/dL). In conjunction with xerostomia and xerophthalmia, these findings were highly suggestive of Sjögren disease (SD) [1]. Gray-scale ultrasonography (US) demonstrated multiple bilateral hypoechoic nodular lesions with a characteristic honeycomb-like architecture throughout both parotid glands (**A**,**B**). Color Doppler examination revealed mild-to-moderate internal vascularity within the lesions (**C**). Real-time elastography demonstrated heterogeneous tissue stiffness with several focal hard areas represented by blue coloration (**D**). Shear wave elastography further confirmed heterogeneous elasticity, with a measured stiffness value of 69.5 kPa in a representative lesion (**E**). To further evaluate the etiology and exclude IgG4-related disease [2] or lymphoproliferative disorders, US-guided core needle biopsy (US-CNB) of the right parotid gland was performed. The arrow denotes the biopsy needle visualized under ultrasound guidance. (**F**) Histopathological examination showed dense chronic inflammatory infiltrates with lymphoepithelial lesions and a mixed population of B and T lymphocytes. No significant increase in IgG4-positive plasma cells was identified. Due to limited tissue sampling, the differential diagnosis included lymphoepithelial sialadenitis and chronic sialadenitis, while extranodal marginal zone B-cell lymphoma (MALT lymphoma) could not be completely excluded.

**Figure 2:**
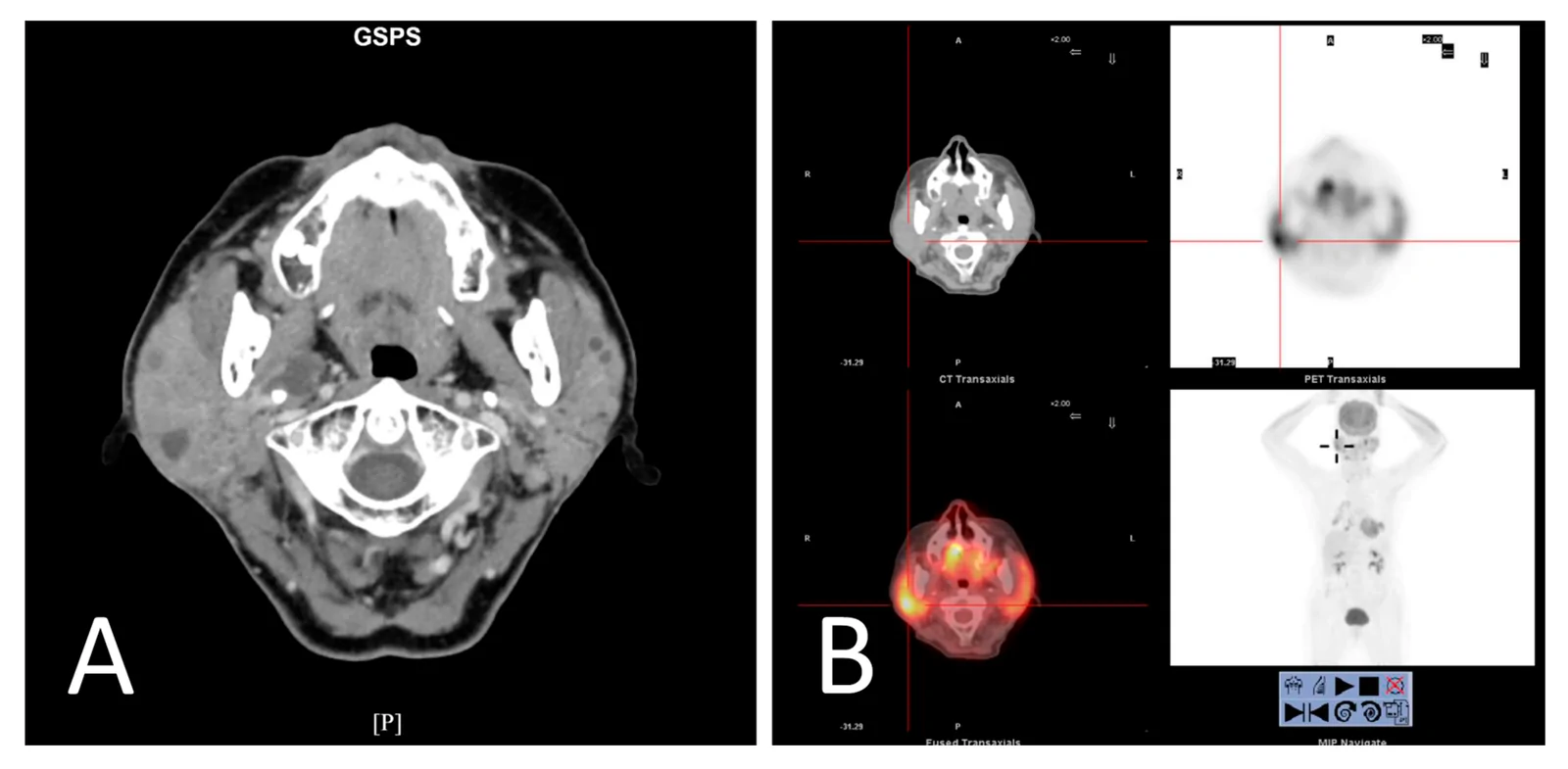
Subsequent neck computed tomography demonstrated bilateral parotid gland enlargement with multiple cystic changes and necrotic lymphadenopathy in the right parapharyngeal space (**A**), raising suspicion for lymphoma. The patient therefore underwent surgical excision of the right parotid lesion for definitive diagnosis. PET-CT demonstrated lymphoma involvement of bilateral parotid glands (**B**), multiple nodal regions above the diaphragm, and pulmonary lesions.

**Figure 3:**
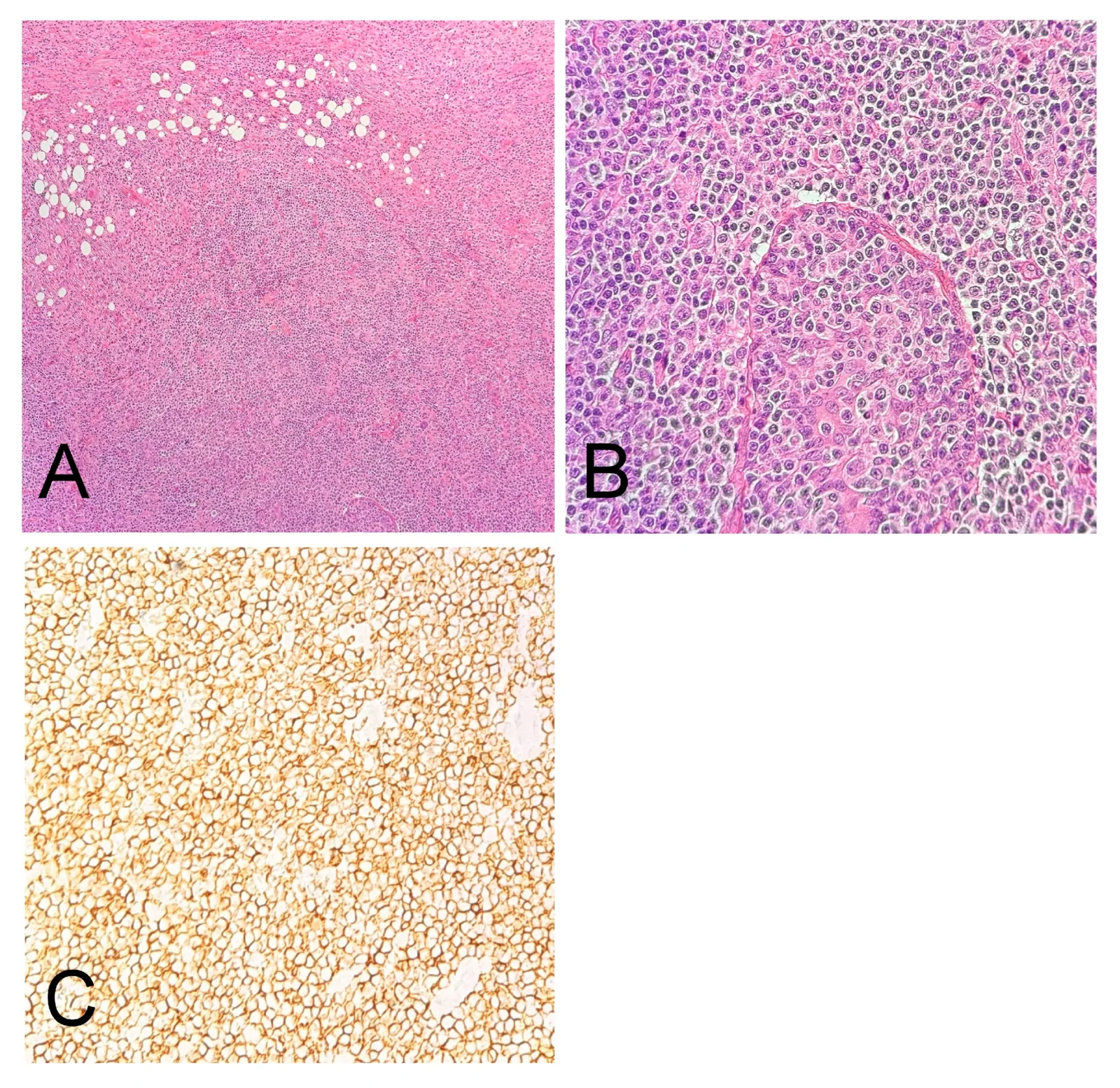
Histopathologic and immunohistochemical findings of the excised parotid gland are shown. Low-power examination showed a low-grade B-cell lymphoma with predominantly nodular and focally diffuse infiltration of small lymphoid cells, extensively involving the salivary gland parenchyma and extending into the adjacent adipose tissue (H&E; original magnification, ×100, (**A**)). High-power examination demonstrated prominent lymphoepithelial lesions formed by neoplastic lymphoid cells infiltrating salivary ductal epithelium (H&E; original magnification, ×400, (**B**)). Immunohistochemical staining showed diffuse membranous positivity for CD20 in the neoplastic B cells (original magnification, ×400, (**C**)). The neoplastic cells were also focally positive for BCL2 and showed marked kappa light-chain predominance over lambda, while they were negative for CD3, CD5, CD10, BCL6, and cyclin D1. CD21 demonstrated disrupted follicular dendritic cell meshworks, cytokeratin highlighted the lymphoepithelial lesions, and Ki-67 showed a low proliferative index. Overall, the morphologic and immunophenotypic findings supported the diagnosis of extranodal marginal zone B-cell lymphoma (MALT lymphoma). Immunoglobulin gene rearrangement analysis was not performed because the diagnosis was considered definitive based on the histologic and immunophenotypic findings. In this case, the presence of multiple hypoechoic nodules in the bilateral parotid glands raised suspicion for either autoimmune sialadenitis or lymphoma, highlighting the importance of tissue diagnosis. US plays an important role as a first-line imaging modality in the evaluation of salivary gland diseases [3]. Both SD-related sialadenitis and parotid MALT lymphoma may present as multiple hypoechoic lesions with heterogeneous parenchymal echotexture on ultrasonography. However, progressive focal enlargement, confluent hypoechoic lesions, or associated cervical lymphadenopathy should raise suspicion for lymphoma. Because substantial imaging overlap exists, imaging findings should be interpreted together with the clinical, serological, and pathological findings. US-CNB has emerged as a valuable diagnostic tool for parotid lesions due to its minimally invasive nature and relatively high diagnostic accuracy [4]. Compared with fine needle aspiration, CNB provides preserved tissue architecture, allowing better evaluation of lymphoid proliferation and lymphoepithelial lesions [5]. However, sampling error may occasionally occur, particularly in heterogeneous diseases such as SD-associated lymphoproliferative disorders. The initial CNB findings likely reflected both limited tissue sampling and the histological overlap between benign lymphoepithelial sialadenitis and early MALT lymphoma. Because both entities may demonstrate dense lymphoid infiltrates and lymphoepithelial lesions, definitive diagnosis can be challenging in small biopsy specimens. In our case, the CNB specimen demonstrated features suggestive of lymphoepithelial sialadenitis but did not exclude MALT lymphoma. This case suggests that benign or inconclusive CNB findings should be interpreted cautiously when clinical suspicion persists. The temporal relationship between Sjögren’s disease and MALT lymphoma remains uncertain. Sjögren’s disease may have been previously unrecognized or clinically asymptomatic, while some features of MALT lymphoma may overlap with Sjögren-associated sialadenitis. This case suggests that ultrasonography, real-time elastography, and CT may have complementary roles in the evaluation of parotid lesions; however, benign or inconclusive CNB findings should be interpreted cautiously when clinical suspicion persists. Repeat biopsy or surgical excision may be considered in selected cases with discordant clinical and imaging findings. These observations are based on a single case and should be considered hypothesis-generating.

## Data Availability

The original contributions presented in this study are included in the article. Further inquiries can be directed to the corresponding author.
